# The Effect of Whey Protein Supplementation on the Temporal Recovery of Muscle Function Following Resistance Training: A Systematic Review and Meta-Analysis

**DOI:** 10.3390/nu10020221

**Published:** 2018-02-16

**Authors:** Robert W. Davies, Brian P. Carson, Philip M. Jakeman

**Affiliations:** 1 Human Science Research Unit, Centre for Interventions in Infection, Inflammation & Immunity (4i), University of Limerick, Limerick V94 T9PX, Ireland; brian.carson@ul.ie (B.P.C.); phil.jakeman@ul.ie (P.M.J.); 2 Food, Health Ireland, Physical Education and Sport Sciences Department, University of Limerick, Limerick V94 T9PX, Ireland

**Keywords:** athletic performance, dietary supplements, exercise, humans, recovery of function, resistance training, skeletal muscle, sports, weight lifting, whey proteins

## Abstract

Whey protein (WP) is a widely consumed nutritional supplement, known to enhance strength and muscle mass during resistance training (RT) regimens. Muscle protein anabolism is acutely elevated following RT, which is further enhanced by WP. As a result, there is reason to suggest that WP supplementation may be an effective nutritional strategy for restoring the acute loss of contractile function that occurs following strenuous RT. This systematic review and meta-analysis provides a synthesis of the literature to date, investigating the effect of WP supplementation on the recovery of contractile function in young, healthy adults. Eight studies, containing 13 randomised control trials (RCTs) were included in this review and meta-analysis, from which individual standardised effect sizes (ESs) were calculated, and a temporal overall ES was determined using a random-effects model. Whilst only half of the individual studies reported beneficial effects for WP, the high-quality evidence taken from the 13 RCTs was meta-analysed, yielding overall positive small to medium effects for WP from < 24 to 96 h (ES range = 0.4 to 0.7), for the temporal restoration of contractile function compared to the control treatment. Whilst the effects for WP were shown to be consistent over time, these results are limited to 13 RCTs, principally supporting the requirement for further comprehensive research in this area.

## 1. Introduction

Resistance training (RT) is used to increase lean mass, strength, and physical function [1]. The ability to sustain high-quality exercise performance during periods of intense training is a key component for optimal and efficient progression. Strenuous RT can evoke damage or deformation to the working muscle, limiting its capacity to produce force—this may persist for hours or days before full recovery, reducing general ‘muscle function’ [2,3]. During any subsequent training/performance bout a reduction in muscle function will impair quality and intensity, and is, potentially injurious to the athlete. Protein ingestion is thought to enhance peri-training recovery of muscle function, but the extant literature is, to date, equivocal. Whey protein (WP) is a high-quality source of protein, rich in essential amino acids, known to enhance muscle protein synthesis (MPS) post-exercise [4,5,6,7] which is demonstrably superior compared to other lower quality protein sources [8]. Because of these properties, WP is purported to accelerate the recovery of muscle function following RT [9,10,11,12,13,14,15,16], though the supporting evidence is far from clear. Evaluation of the evidence for the efficacy for WP may be confounded by experimental factors such as RT mode, subjects’ training status, outcome measures, and supplementation strategy. The aim of this systematic review and meta-analysis is to examine and synthesise the current evidence from high-quality randomised control trials (RCTs) in the extant literature, to determine the effect of WP on the recovery of muscle function following RT, in young, healthy adults.

## 2. Materials and Methods

This systematic review and meta-analysis was conducted and reported in accordance with the recommendations of the Preferred Reporting Items for Systematic reviews and Meta-analysis (PRISMA) guidelines [17].

### 2.1. Eligibility Criteria

Studies meeting the following criteria were considered for review and meta-analysis: (1) subjects were healthy, human, adults; (2) the study contained at least two treatments, where at least one treatment was a WP and another was a placebo or control treatment; (3) subjects were randomly assigned to each group or treatment; (4) some form of muscle function was measured (defined below); (5) function was assessed following a RT intervention only (defined below); (6) original research published in peer reviewed indexed journals; and (7) written in English.

Muscle function following RT was analysed as the principal outcome measure. Muscle function was operationally defined as (1) peak force or torque produced during an isometric, isokinetic, or isoinertial maximal voluntary contraction (MVC); or (2) the maximum load that could be lifted, repetitions, or work done during a standardised exercise task. RT was operationally defined as an any exercise consisting of repeated muscle actions opposed by an external (exogenous) force.

Studies were excluded from the review and meta-analysis if: (1) the WP supplement was co-ingested with any other ergogenic aid(s) evidenced to enhance muscle function (e.g., caffeine [18], β-hydrolxy β-methylbutyric acid [19], branched chain amino acids [20], or anti-inflammatory drugs, supplements or analgesics [21]); (2) insufficient data was reported to conduct statistical analysis and/or to confirm eligibility; (3) any other temporal therapeutic intervention was conducted during the recovery period (e.g., massage, compression, exercise, heat therapy etc.); and/or (4) data had been duplicated or reported elsewhere.

### 2.2. Search Strategy and Study Selection

A computerised literature search was performed (September 2017 to December 2017) using four online databases: Medline (Pubmed), Web of Science, Science Direct, and the Cochrane library. Title/Abstract/Keyword searches were made using Boolean search operators (“whey protein” or “protein supplement”) and (“exercise” or “force” or “function” or “recovery” or “resistance exercise” or “strength training” or “torque” or “weight lifting”). A supplementary search of keywords was made, as were reference lists and citations of all identified studies, using Google Scholar.

A total of *n* = 930 studies were identified from the databases and other supplementary searches. After adjusting for duplicates *n* = 399 studies remained. During screening *n* = 354 were removed following title/abstract review, not meeting eligibility criteria (see above). The full text of the remaining *n* = 45 studies was examined in detail; *n* = 37 were excluded (*n* = 14, reporting no measure of acute function; *n* = 1, not written in English; *n* = 1, absence of statistical data; *n* = 3, not RT; *n* = 11, no WP intervention or control; *n* = 5, WP supplement was co-ingested with other ergogenic supplements; *n* = 2, data duplicated or previously reported). In total *n* = 8 studies were included for the systematic review and meta-analysis [9,10,11,12,13,14,15,16] (Table 1), meeting the predefined eligibility criteria set by the authors (see above).

### 2.3. Data Extraction

Data pertaining to subjects’ characteristics (sex and training status), experimental design (crossover/parallel, blinding, randomisation), function measure (type, time-point, muscle group), level of muscle damage (circulating creatine kinase (CK) level), RT intervention (load, volume, duty cycle, muscle action, muscle group), and supplementation strategy (dietary control, supplement control, dose, timing, type) was noted and reported (Table 1). For analysis, mean, SD, and sample size (*n*) were extracted (available from all included studies) from the treatment and control groups for each variable. These data were reported as either the post-treatment measurement in standard units, or as a percentage change pre-treatment to post-treatment.

## 3. Analysis

### 3.1. Study Quality

Study quality was categorised using the Quality Criteria Checklist for Primary Research to avoid bias [22] (Table 1). Briefly, studies were assessed on: (1) the research question being stated; (2) subject selection being free from bias; (3) compatible treatment groups; (4) method for withdrawals described; (5) blinding; (6) intervention details reported; (7) outcome(s) reported and valid; (8) appropriate statistical analysis; (9) appropriate conclusions stated and limitations cited; and (10) funding/sponsorship was free from bias. Items 2, 3, 6, 7, plus one other item was required for a positive rating (+), a neutral rating (ο) was assigned if any of these items were not met, and a negative rating (−) was given if six or more items were not reported. 

### 3.2. Meta-Analysis

For the review and meta-analysis, from the eight included studies multiple independent (parallel) treatment groups were conducted in four studies [9,12,13,16], which were analysed as independent RCTs (13 in total). The individual temporal effects (*k*) within each RCT were identified as being >3 h post-treatment [4], and for inclusion it was a precondition that a reduction in muscle function was observed in the control treatment; where no dysfunction occurs, no treatment effect can be observed.

Peak isometric knee extensor strength was used as the criterion functional measure for the meta-analysis, as it was (1) measured within all included studies reducing heterogeneity between effects; (2) the largest *k* value amongst all reported functional measures (see definition above); and (3) in the extant literature the knee extensor muscles are a widely assessed/examined muscle group, and the isometric MVC is a criterion measure of strength/function holding high internal and external validity.

For the meta-analysis Hedges *g* effect size (ES) was calculated for each effect by a pooled SD for both parallel and crossover designs, using the reported *∆* values from all RCTs (i.e., % change pre-treatment to post-treatment) [23]. Heterogeneity was assessed for each time-point by Cochran’s Q (*Q*) and *I*^2^ (<24 h, *k =* 8, *Q* = 20.6; *p* = 0.004; *I*^2^ = 66%; 24 h, *k =* 13 *Q* = 29.8; *p* = 0.003, *I*^2^ = 60%; 48 h, *k =* 7 *Q* = 8.0; *p* = 0.241, *I*^2^ = 24%; 72 h, *k =* 7 *Q* = 20.2; *p* = 0.003, *I*^2^ = 70%; 96 h, *k =* 7 *Q* = 14.0; *p* = 0.030, *I*^2^ = 57%) [24]. As ‘small/moderate’ heterogeneity was observed between ESs, a random effects model was used to calculate the pooled ESs which are reported with (lower, upper) 95% CI [23] (R Studio 1.1.383). For temporal analysis, effects were collapsed into 24-h epochs (<24, 24, 48, 72 and 96 h). A meta-regression and subgroup analysis was used to determine temporal effects and the effect of the experimental approach (SPSS 24). ESs are interpreted as 0.2 = ‘small’; 0.5 = ‘medium’; 0.8 = ‘large’ ES [25].

## 4. Results

### 4.1. Study Quality and Content

All studies were positively ranked being of reasonably high quality. Predefined eligibility criteria were reported, control groups with random group allocation were used throughout, and appropriate experimental design/control/analysis/reporting was completed for all studies. All but one study [13] used a placebo as a control, in either a blind or double-blind experiment. Funding source(s), and a declaration of conflict/competing author interests(s) was reported in all but one study [16]. In total, 152 subjects were used from the eight included studies, consisting of 13 RCTs, with 42 temporal ESs. One study tested young, healthy women in a mixed-sex sample [12] with the remaining seven studies (~92% of subjects) testing young, healthy men.

### 4.2. Muscle Function

**Figure 1:**
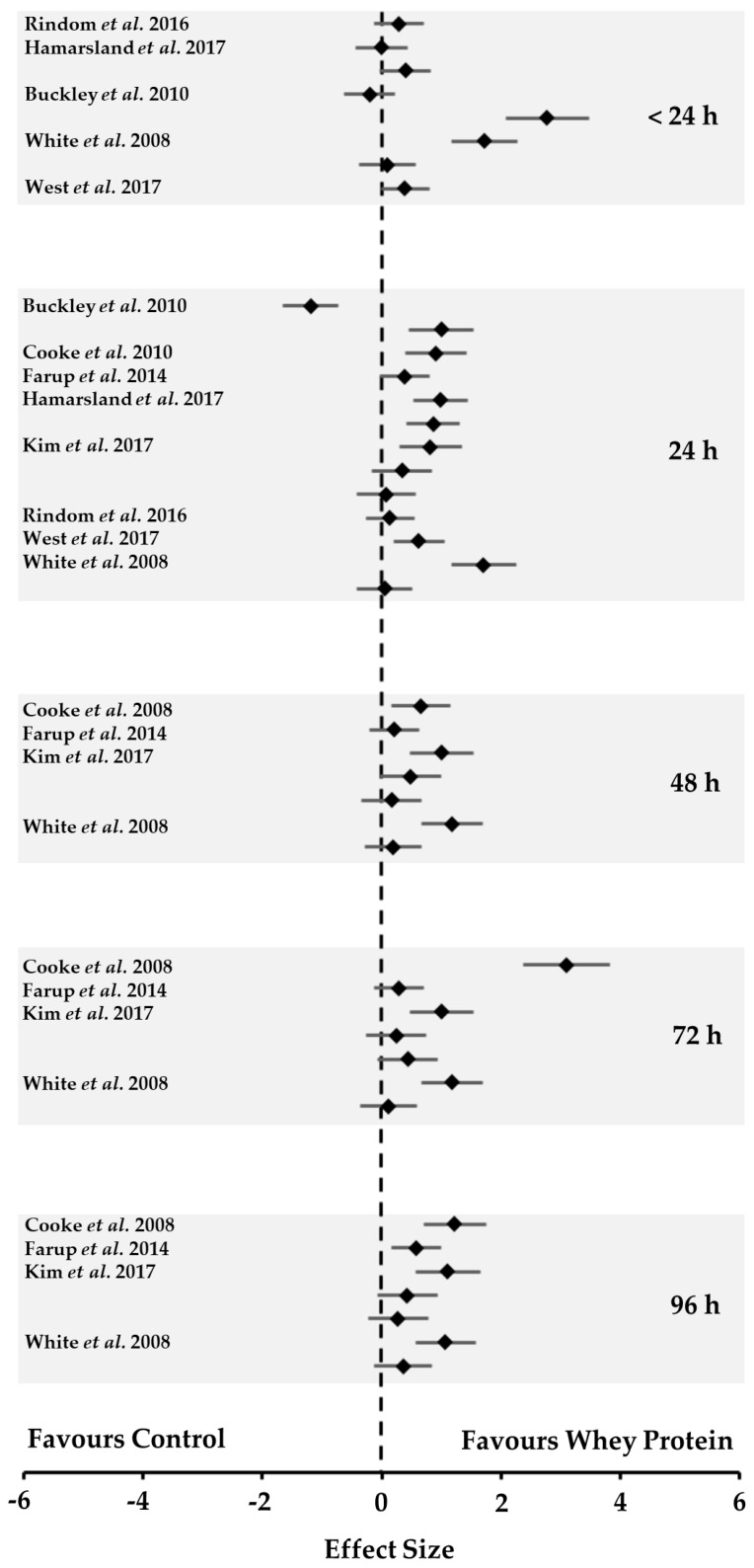
Forest plot of the temporal effect sizes (ESs) of the whey protein supplement for the recovery of muscle function following resistance training, compared to a control treatment. Data are mean ± 95% CI. ESs are ordered by time.

Four of the included studies reported overall beneficial effects for WP compared to the control treatment [9,10,15,16]. No overall effect for WP was reported in the remaining four studies [11,12,13,14]. Only one negative temporal-treatment effect was reported [9], with ESs ranging widely within and between studies (−1.1 to 4.8) (Figure 1). From the meta-analysis, small to moderate effects were observed over time at <24 h (ES = 0.6 [0.1, 1.0], *z* = 2.00, *p* = 0.046); 24 h (ES = 0.4 [0.1, 0.8], *z* = 1.83, *p* = 0.067); 48 h (ES = 0.4 [−0.1, 0.9], *z* = 1.35, *p* = 0.176); 72 h (ES = 0.7 [0.2, 1.3], *z* = 1.35, *p* = 0.025) and 96 h (ES = 0.4 [−0.1, 0.9], *z* = 1.36, *p* = 0.174). No moderator effect was observed for time (*β* = 0.001, *z* = 0.677, *p* = 0.498). 

### 4.3. Supplementation Strategy 

For all studies, WP was administered in a single bolus, the median dose being 25 g (range = 20 g to ~120 g). Sixty-two percent of the RCTs administered the WP bolus post-exercise, 23% pre-exercise, and 15% of treatments ingested WP pre- and post-exercise. Repeated WP boluses were given throughout the temporal recovery period in all but two studies [13,16], half of these studies gave subjects multiple boluses per day, and half administered WP at 24 h intervals throughout the temporal recovery period. Outside of the supplement dosing strategy, dietary intake was controlled in three studies, providing adequate and standardised dietary protein intake for all subjects [12,14,15]. The subjects in four of the remaining studies were asked to maintain their normal dietary intake, which was reported in two of these studies. Dietary information was provided in all but one study [9], but no information pertaining to subjects’ individual dietary intake was reported. In lieu of the WP treatment a placebo/control drink was provided in all bar one study. An isocaloric CHO drink was the most common placebo/control in 3 out of the 8 studies. Other proteins low in essential amino acids (collagen), water, and mixed-macronutrient drinks (milk) were also used for the placebo/control treatment.

### 4.4. Resistance Training Intervention 

Seventy percent of the RT interventions used eccentrically biased muscle actions at forces greater than one maximum repetition (1 RM) or peak isometric force (P_o_)—selecting untrained subjects for this RT intervention. The remaining studies used trained subjects undertaking performance or traditional RT (i.e., isoinertial < 1 RM/P_o_). Small to medium overall ES were noted for both experimental approaches (untrained ES = 0.5 [0.3, 0.7]; trained ES = 0.4 [−0.1, 0.9]). Five studies measured the activity of a soluble enzyme released from the muscle, creatine kinase (CK), which is an indirect marker of exercise-induced muscle damage (EIMD). CK was not assessed further than 24 h in any of the performance-based RT interventions [12,14,15]. Following the eccentrically biased RT, CK activity was elevated for up to 96 h in four studies [10,11,13,16], and no increase was observed in the one remaining study (measured at 24 h only) [9]. Temporal changes in muscle soreness correlated with CK, remaining elevated above baseline up to 96 h in all five of the eccentrically biased RT studies. No temporal beneficial effect for WP was observed for CK activity or muscle soreness in any study. 

## 5. Discussion

Overall small to medium ergogenic effects were observed for WP supplementation restoring muscle contractile function following RT from < 24 to 96 h (ES range = 0.4–0.7). Half of the included studies reported a beneficial overall effect for WP, whilst the other half concluded no effect occurred compared to the control. No study reported any overall negative effect for WP and only one negative effect (favouring the control) was reported out of the 44 temporal comparisons. Although the RCTs were of reasonably high quality, significant heterogeneity was observed between studies.

### 5.1. Experimental Approaches to the Problem

A clear break point was identified between two experimental approaches. A dichotomy was observed for the selection of (1) subject population (i.e., trained or untrained); (2) muscle action (i.e., eccentric only or concentric + eccentric); and (3) load (<or >1 RM/P_o_). In five studies it is clear that one experimental approach was used to evoke overt EIMD, dysfunction and/or soreness [9,10,11,13,16], however, a ‘performance-based’ approach was employed in the remaining three more recent studies [12,14,15]. Whilst there are fundamental differences between these two approaches, similar effects were observed for WP. On review, no consensus on the efficacy of WP was attained in either subgroup.

Similar, contrasting results have been observed in the wider extant literature, following alternative forms of damaging exercise [26,27,28], and/or high-protein mixed macronutrient [29,30,31,32,33,34,35], performance-based exercise interventions [29,36], or within a more general systematic review [37]. There was no definitive evidence to suggest that WP reduced markers of EIMD or muscle soreness following RT. Whilst this is the first review quantifying overall temporal ES, the constituent studies are biased towards purposefully damaging exercise. Other than more recent investigations [12,14,15], generally, performance-based exercise interventions are rarely used limiting the ecological and external validity of these findings.

Outside of the criterion functional measure of peak isometric torque, two studies conducted additional performance-based testing in line with the RT intervention (i.e., countermovement jump, RT performance, and anaerobic performance) [14,15]. Although similarities were noted between studies for the sample-population, supplement strategy, outcome measures and RT intervention, contrasting results were reported, requiring further conclusive research. 

### 5.2. Supplementation Strategy 

WP is a widely researched and well characterised supplement with known bioactivity and biokinetics. The amount of WP required to optimise post-exercise anabolism approximates to 20 g [4,7], which was achieved in all 13 RCTs. The timing of the protein feed varied, and whilst pre-exercise ingestion is shown to enhance post-exercise recovery (c.f. post-exercise ingestion [16]), these findings were not consistent, with the timing of WP ingestion also not shown to effect post-exercise recovery [13]. Repeated WP doses were administered throughout the temporal recovery period in all but two studies, either as repeated daily doses or at 24 h intervals. The amount of protein ingested from the WP supplement constituted only a fraction of the total dietary protein intake during the period of recovery. As dietary control was absent in 70% of the RCTs, the influence of dietary protein source, quantity and distribution between subjects and treatment groups is a further confounder to the outcome, and potentially the greatest source of variation between the observed effects, which is highlighted as a major limitation in the extant literature. 

In addition to the considerations above, a variety of placebos/controls were utilised in the RCTs. Whilst effects have been observed for WP compared to neutral (non-anabolic) controls (i.e., CHO and water [4]) null effects were generally reported for studies using lower-quality protein supplements or milk as a control [30,32,33]. It is possible that potential bioactivity of any placebo/control could ameliorate the treatment effect of the WP, a potential confounder to the outcome. 

### 5.3. Other Considerations

Considering the high prevalence of WP consumption amongst people undertaking regular RT [38], it was surprising that only eight high-quality studies have been published over a period of 10 years. The authors noted a similar number of eligible studies were reported within the grey literature, which for quality control were not included in this review and meta-analysis. An obvious sex bias was also observed, with the authors identifying the need for further high-quality research—specifically directed toward the performance-based experimental approach (viz. trained subjects, performance-based RT/functional measures, repeated bouts). Additionally, whilst empirically advanced by some [11,14,15,39], in the absence of integrative or confirmative evidence supporting a physiological mechanism(s) that adequately explains the acute loss of muscle function following RT, the promotion of a protein-supplement based strategy to enhance the recovery of function is limited. 

## 6. Conclusions

This systematic review identified eight individual studies consisting of 13 RCTs that satisfied the criteria for inclusion in the meta-analysis. A small to medium temporal ergogenic effect was observed for WP, accelerating the recovery of muscle function following RT, with under half of the RCTs reporting beneficial overall effects for WP. The findings from this systematic review and meta-analysis are limited to eight studies, and considered underpowered for further analysis/control of other extraneous and/or potentially confounding factors such as the subject characteristics, mode/purpose of exercise, and/or the supplementation strategy. Authors caution against interpreting the overall temporal ES reported in this study as definitive evidence. Rather, it is suggested these findings are understood as a synthesis of the literature to date, reflecting the dispersion, limitations, and overall effects between the RCTs in the extant literature, identifying the need for further experimental research in this area. 

## Figures and Tables

**Table 1:** Studies included in the systematic review and meta-analysis.

Study	Subjects	Design	QA	Exercise	Load	Supplement(s)	Control	Dose and Timing	Function Measure
Buckley et al. 2010 [9]	Untrained men	Parallel Groups	+	Unilateral, isokinetic, eccentric, knee extensions	100 MVCs no rest reported	WP isolate (*n =* 11) WP hydrolysate (*n =* 6)	Flavoured Water (*n =* 11) No dietary control	25 g 0 h, 6 h, 22 h post-exercise	Peak isometric knee extensor strength
Cooke et al. 2010 [10]	Untrained men	Parallel Groups	+	Unilateral, eccentric knee extensions, flexions, leg presses	4 sets × 10 reps 120% 1 RM 3 min rest each set	WP hydrolysate + CHO (9:1) (*n =* 9)	Isocaloric CHO (*n =* 8) No dietary control	1.5 g/kg 0.5 h post-exercise plus ~30 g with meals each day	Isometric and isokinetic knee extensor and flexor strength
Farup et al. 2014 [11]	Untrained men	Parallel Groups	+	Unilateral, isokinetic, eccentric knee extensions	15 sets × 10 MVCs 1 min rest each set	WP hydrolysate + CHO (1:1) (*n =* 12)	Isocaloric CHO (*n =* 12) No dietary control	56 g 0 h, 24 h, 48 h post-exercise	Peak isometric knee extensor strength
Hamarsland et al. 2017 [12]	Trained men and women	Parallel Groups	+	Bilateral knee extensions leg presses	4 sets × 8 reps 100% 8 RM 3 min each set	WP concentrate (*n =* 10) Native WP (*n =* 10)	Milk (*n =* 12) Fixed dietary control	20 g 0 h and 2 h post-exercise	Peak isometric knee extensor strength
Kim et al. 2017 [13]	Untrained men	Parallel Groups	+	Bilateral, isokinetic eccentric elbow flexions	2 sets × 25 MVCs	WP (*n =* 24) (3 groups × *n =* 8)	No placebo/control No dietary control	1.5 g/kg. Immediately before or after or before & after exercise	Peak isometric elbow flexor strength
Rindom et al. 2016 [14]	Trained men	Crossover	+	Whole body 5 exercises	3–5 sets × ≤ 12 reps per exercise, 100% 15 RM 1.5 min rest each set	WP (*n =* 12)	Collagen protein (*n =* 12) Fixed dietary control	25 g immediately before and after exercise 24 & 48 h post-exercise	Peak isometric knee extensor and flexor strength, CMJ, 30 s Wingate test, 3 RMs
West et al. 2017 [15]	Trained men	Crossover	+	Whole body 6 exercises	4 sets × 8 reps per exercise, 75% 1 RM 2 min rest each set	WP isolate, concentrate and peptides (*n =* 12)	Isocaloric CHO (*n =* 12) Fixed dietary control	25 g 0 h and 10 h post-exercise	Peak isometric knee extensor strength and maximum reps at 75% 1 RM, CMJ, 30 s Wingate
White et al. 2008 [16]	Untrained men	Parallel Groups	+	Unilateral, isokinetic, eccentric knee extensions	5 sets × 10 MVCs 1 min rest each set	WP + CHO (1:3) (*n =* 18) (2 groups × *n =* 9)	Flavoured water (*n =* 9). No dietary control	98 g Immediately before or after exercise	Peak isometric knee extensor strength

CHO = carbohydrate; CMJ = countermovement jump; MVC = maximal voluntary contraction; QA = quality assessment; RM = repetition maximum; WP = whey protein.
